# Marked variation between winter and spring gut microbiota in free-ranging Tibetan Macaques (*Macaca thibetana*)

**DOI:** 10.1038/srep26035

**Published:** 2016-05-16

**Authors:** Binghua Sun, Xi Wang, Sofi Bernstein, Michael A. Huffman, Dong-Po Xia, Zhiyuan Gu, Rui Chen, Lori K. Sheeran, R. S Wagner, Jinhua Li

**Affiliations:** 1 School of Resources and Environmental Engineering, Anhui University, Hefei, China; 2 Primate Research Institute, Kyoto University, Japan; 3 School of Life Sciences, Anhui University, Hefei, China; 4 Department of Anthropology, Central Washington University, Ellensburg, Washington, USA; 5 Department of Biological Sciences and Primate Behavior Program, Central Washington University, USA; 6 School of Life Sciences, Hefei Normal University, Hefei, China

## Abstract

Variation in the availability and distribution of food resources is a strong selective pressure on wild primates. We explored variation in Tibetan macaque gut microbiota composition during winter and spring seasons. Our results showed that gut microbial composition and diversity varied by season. In winter, the genus *Succinivibrio*, which promotes the digestion of cellulose and hemicellulose, was significantly increased. In spring, the abundance of the genus *Prevotella*, which is associated with digestion of carbohydrates and simple sugars, was significantly increased. PICRUSt analysis revealed that the predicted metagenomes related to the glycan biosynthesis and metabolic pathway was significantly increased in winter samples, which would aid in the digestion of glycan extracted from cellulose and hemicellulose. The predicted metagenomes related to carbohydrate and energy metabolic pathways were significantly increased in spring samples, which could facilitate a monkey’s recovery from acute energy loss experienced during winter. We propose that shifts in the composition and function of the gut microbiota provide a buffer against seasonal fluctuations in energy and nutrient intake, thus enabling these primates to adapt to variations in food supply and quality.

Survival and reproduction are dependent upon each individual’s ability to adjust to seasonal variation in food availability, abundance and distribution^1^. As one moves away from the equator, photosynthesis, plant growth and reproduction are increasingly affected by seasonal variation^2^. Animal patterns of nutrient intake change dramatically across seasons and habitats due to variation in temporal availability of food resources^3^. Understanding how wild animals solve these feeding challenges provides insight into the ecology and evolution of species^4^.

Non-human primates (NHPs) originated in tropical rainforest habitats and radiated into woodland, savanna and montane environments^5^. To adapt to temporal fluctuations in food resources while meeting nutritional requirements needed to sustain life, they have evolved anatomical and behavioral strategies, including specialized stomachs, habitat use patterns, ranging behavior and social organization^6^,^7^,^8^,^9^,^10^. Generally, NHPs prefer high-quality foods such as fruits and young leaves, which are rich in easily-digestible carbohydrates, lipids and proteins. However, these foods are not consistently available throughout the year^5^,^9^,^11^,^12^. For example, in winter, NHPs living in temperate environments eat a wide variety of low-quality foods^13^. Low-quality foods like mature leaves, gum and roots are often high in cellulose and dietary fiber which are difficult to digest^14^,^15^. Previous studies have found that primates shift their activity budgets and foraging patterns in response to ecological pressures imposed during periods of food scarcity^9^,^16^,^17^,^18^. How wild primates digest and metabolize low-quality foods during periods of limited food resource availability has been overlooked^3^.

The mammalian digestive system is home to a complex microbial community, many of which have symbiotic relationships with particular hosts^19^,^20^,^21^,^22^. Studies of humans and laboratory animals have demonstrated that the changes in host diet and physiology can alter gut microbiome composition^20^,^23^,^24^,^25^. Additionally, it has been found that gut microbial communities have mutualistic relationships with host by impacting the host’s ability to digest and assimilate food, thus providing the host with energy and nutrients^24^. For example, gut microbes can help a host break down otherwise indigestible materials such as cellulose and hemicellulose, thereby using these compounds to meet physiological needs^26^. Changes in gut microbiome composition are therefore generally considered to be microbial responses to selective pressures imposed by changes in the host’s diet, health and physiology^21^,^22^. These microbial shifts allow the host to digest food items more efficiently to meet energy and nutrient demands^3^,^27^. Mutualistic host/microbe relationships likely vary seasonally in response to temporal alterations in food resources^3^, and this response is expected to be particularly apparent at sites with distinct seasonal variation in the occurrence of high- and low-quality foods. However, few data are currently available to explore this relationship in wild primates.

Our study population of free-ranging Tibetan macaques (*Macaca thibetana*) is ideal to explore seasonal variation in gut microbiota. The members of our study group (Yulinkeng A1 or YA1) have been observed for nearly 30 years. All adult group members can be identified by distinctive physical features (i.e. scars, hair color patterns and/or facial/body appearance), and the ages and matrilineal relationships of all YA1 monkeys are known. Male immigrants’ ages are estimated from comparisons to males of known ages, and their identities are thereafter monitored by their distinctive physical features (i.e. missing canines, scars, missing hands/feet)^28^. The troop inhabits deciduous and evergreen broad-leaf mixed montane forests. Previous studies indicated that in general the Tibetan macaque diet consists of leaves, fruits, grass, and to a lesser extent, flowers, roots, and insects^29^,^30^. The site has an annual average temperature of 15.3 °C (highest: 34.2 °C, lowest: −13. 9 °C). Winter temperatures drop below 0 °C. Most winters, the temperature can remain below freezing for more than 40 consecutive days^29^.

We used high-throughput sequencing methods to compare gut microbiota variation between seasons of low- and high-quality food consumption in our study group. From winter to spring, monkeys in the study group experience a seasonal shift in food quality. Temperature and rainfall, which are comparatively homogeneous within seasons, vary more markedly between seasons^29^ thereby affecting food availability. Mature leaves, roots and other fibrous foods with high proportions of cellulose increase in the Tibetan macaques’ winter diet, while the amount of dietary pectin and simple sugars in the diet increases in the spring as monkeys shift their diet to include more young leaves^7^,^29^,^30^,^31^. Based on this seasonal dietary shift, we tested two hypotheses:

1. H1: Gut microbiome diversity, as measured by alpha and beta diversity, differs significantly in winter and spring.
2. H2: The relative abundance of gut microbiome genera that aid in digestion of cellulose and fiber is higher in winter compared to spring, and the relative abundance of genera that aid in digestion of pectin and simple sugars is higher in spring compared to winter.

## Results

### Microbial community profiles

**Figure 1:**
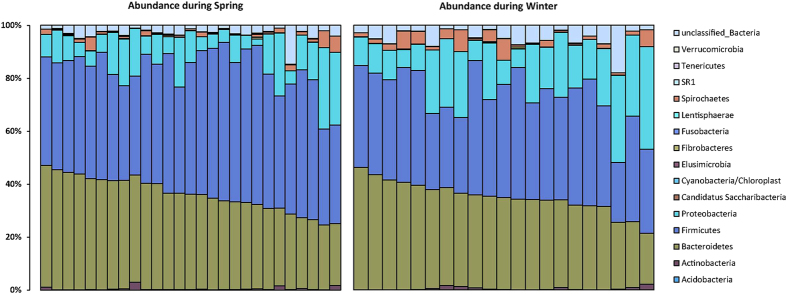
Relative abundance of gut bacterial taxa at the phylum-level. Stacked bar graphs illustrate the abundances of phyla.

After quality filtering, we acquired 572,564 high-quality filtered reads, corresponding to 11,928 ± 2930 reads per monkey from a total of 48 fecal samples. Taxonomic assignment revealed representatives from 14 known bacterial phyla at 97% sequence identity across winter and spring seasons (Fig. 1). Similar to other mammals, Tibetan macaque gut microbiota was largely dominated by Firmicutes (x = 43.43 ± 8.51%), Bacteriodetes (x = 35.20 ± 6.39%) and Proteobacteria (x = 14.67 ± 8.78%). Other represented phyla were Spirochaetes (x = 2.03 ± 2.25%), Actinobacteria (x = 0.48 ± 0.62%) and Verrucomicrobia (x = 0.10 ± 0.079%) (Supplementary Table S2). The predominant genera of bacteria isolated from the monkeys’ fecal samples were *Prevotella* (x = 17.82 ± 0.0867%) and *Succinivibrio* (x = 10.84 ± 0.869%). To evaluate gut microbial diversity, we estimated of diversity within (alpha) gut microbial communities using Shannon diversity index of diversity (H = 4.84 ± 0.39), Simpson’s diversity index (D = 0.038 ± 0.0235), OTU richness (x = 1241 ± 409), Chao 1 (x = 5620 ± 3425) and ACE (x = 3288 ± 1550) (Supplementary Table S1).

### Variation of gut microbiome in winter and spring

To explore gut microbial community differences between seasons and the potential influence of a monkey’s sex and age, alpha diversity including Shannon diversity index, OTU richness, Chao 1 and ACE was estimated with linear mixed models. We found no evidence for significant influence of season or monkey’s sex and age using OTU richness, Chao, ACE (P > 0.1) and Simpson’s diversity index (P = 0.065). However, Shannon diversity index had a significant seasonal difference, whereby species diversity significantly increased in spring samples (Estimate = 0.222, Std. Error = 0.106, df = 31.5, t = 2.09, P = 0.045) compared to winter samples.

**Figure 2:**
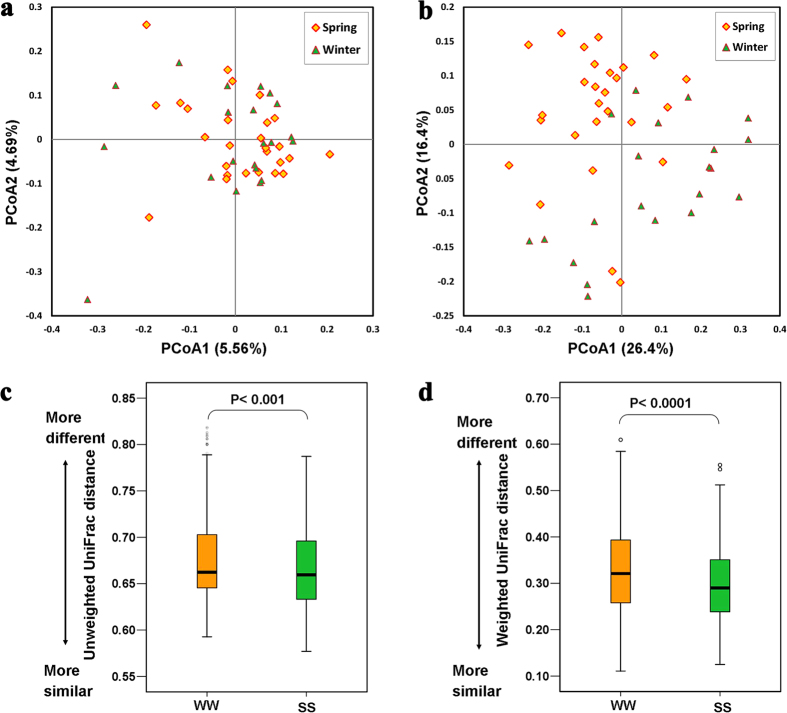
Gut microbiota structure differentiation and inter-individual similarity. PCoA was used to show patterns of separation by season, Yellow: spring sample; green, winter sample; (**a**) based on unweighted UniFrac distance (Permanova, P < 0.05), (**b**) based on weighted UniFrac distance (Permanova, P < 0.01). Permutation test was used to test inter-individual similarity; Yellow box (winter vs winter, WW); green box (spring vs spring, SS); (**c**) based on unweighted UniFrac distance (WW vs SS, permutations = 999, P < 0.001); (**d**) based on weighted UniFrac distance (WW vs SS, permutations = 999, P < 0.0001).

The distribution of beta diversity measures (weighted and unweighted UniFrac distances) was compared between different seasons (winter–winter dyads, WW, N = 210; spring–spring dyads, SS, N = 351, and winter–spring dyads, WS, N = 567). PCoA was used to show patterns of separation by seasons. Permanova tests between WW and SS revealed a significant seasonal separation based on unweighted UniFrac distances (Permanova tests, F = 2.98, P < 0.05) (Fig. 2a) and weighted UniFrac distances (Permanova tests, F = 3.65, P < 0.01) (Fig. 2b). However, we did not find a significant influence (Permanova tests, P > 0.05) of monkeys’ age or sex on UniFrac distances (weighted and unweighted). According to *Costello, E. K. et al.*^32^, we used the UniFrac distances to estimate inter-individual differences within each season. To detect the differences of UniFrac distances (weighted and unweighted) within each season’s samples, we performed permutation tests (permutations = 999) by permuting the W and S labels across individuals and generating a null distribution of mean difference in beta-diversity across the two seasons. A significantly higher dissimilarity was found within winter samples than was found within spring samples (weighted: WW vs SS, P < 0.001; unweighted: WW vs SS, P < 0.0001) (Fig. 2c,d).

**Figure 3:**
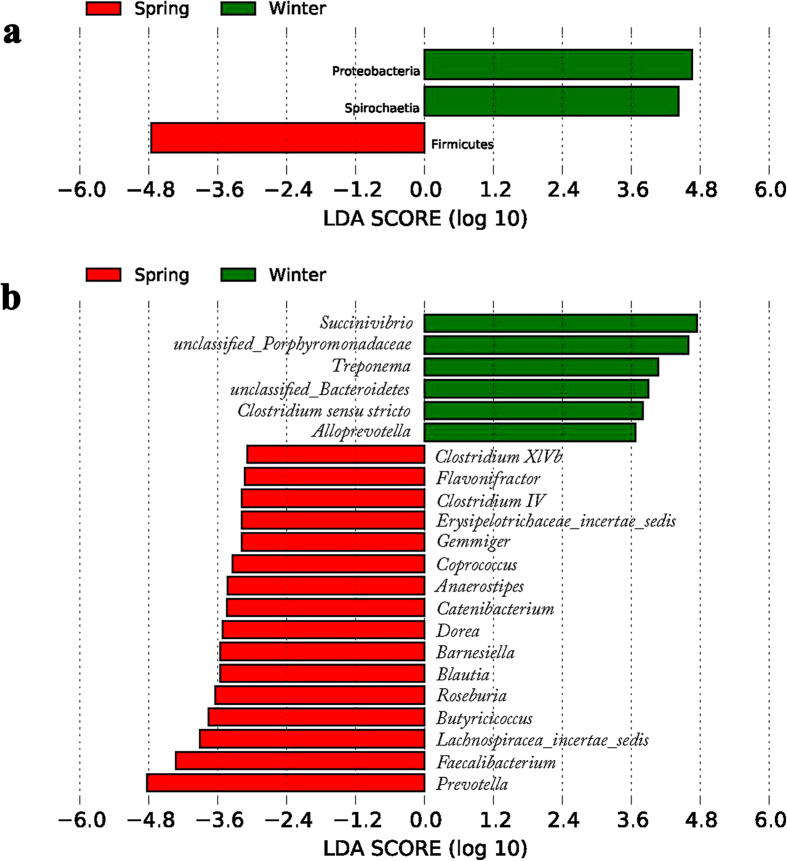
Phylum and genus differentially represented between winter and spring samples identified by linear discriminant analysis coupled with effect size (LEfSe) (LDA > 2, P < 0.05). Red box: enriched in spring samples, green box: enriched in winter samples. (**a**) based on the relative abundance of phylum; (**b**) based on the relative abundance of genus.

To explore the variation of the microbial community composition between the two seasons’ samples, we performed LEfSe tests to detect differences in relative abundance (average relative abundance >0. 01%) of bacterial taxa (including phylum and genus) across samples (Supplementary S2, S3). At phyla levels, our LEfSe analysis revealed that the phyla Proteobacteria and Spirochaetia were significantly enriched in winter samples, while the phylum Firmicutes was significantly enriched in spring samples (LDA > 2, P < 0.05) (Fig. 3a). At the genus level, four known genera (*Succinivibrio, Treponema, Clostridium sensu stricto* and *Alloprevotella*) were significantly enriched in winter samples and sixteen genera (*Prevotella, Blautia, Gemmiger, Barnesiella, Lachnospiracea incertae sedis, ClostridiumXlVb, Anaerostipes, Catenibacterium, Flavonifractor, Coprococcus, Butyricicoccus, Roseburia, Clostridium IV, Dorea Erysipelotrichaceae incertae sedis* and *Faecalibacterium*) were significantly enriched in spring samples (LDA > 2, P < 0.05) (Fig. 3b).

### Variation of predicted metagenomes between winter and spring

**Figure 4:**
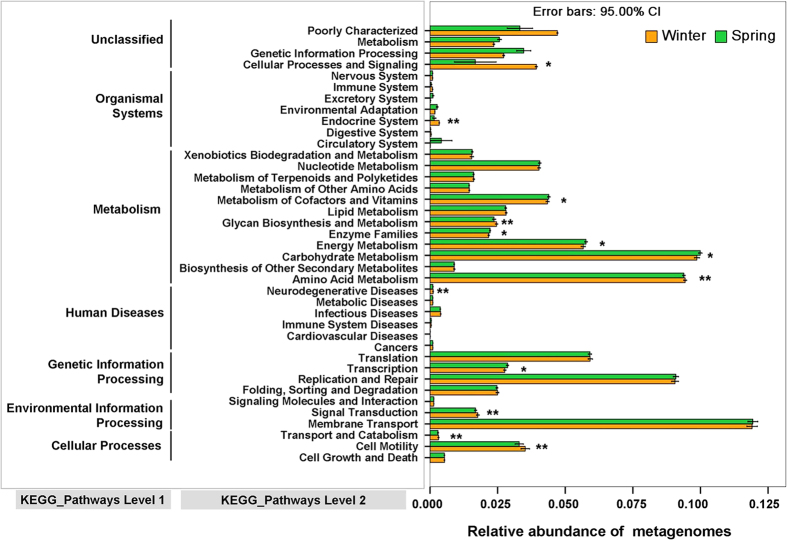
Relative abundance of the predicted gene of metagenome related to KEGG pathways at level 1 and 2; orange box: winter samples, green box: spring samples. The terms given on the left are KEGG pathways annotation at level 1 and level 2 (from left to right). **Enriched in winter samples (LDA > 2, P < 0.05); *enriched in spring samples (LDA > 2, P < 0.05).

**Figure 5:**
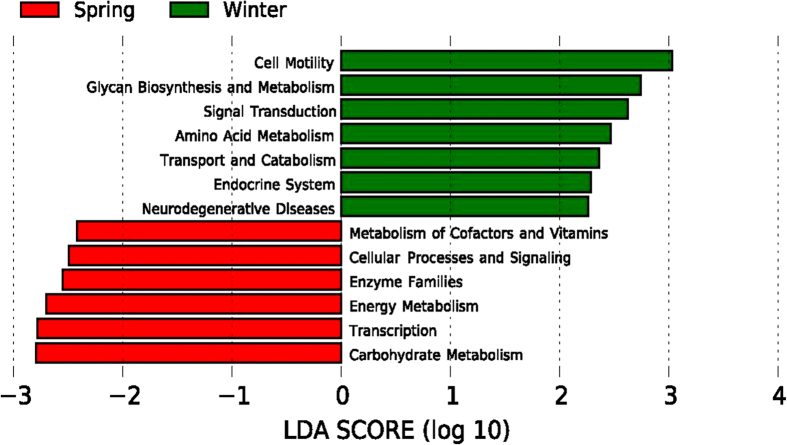
Predicted genes from metagenome related to KEGG pathways differentially represented between the winter and spring samples identified by linear discriminant analysis coupled with effect size (LEfSe) (LDA > 2, P < 0.05). Red box: enriched in spring samples, green box: enriched in winter samples.

Using PICRUSt as a predictive exploratory tool, 39 level 2 KEGG Orthology groups (KOs) were represented in the data set (Fig. 4). The mean weighted nearest sequenced taxon index (NSTI) for our samples was 0.142 ± 0.027. This result is similar with previously reported analyses in mammals (mean NSTI = 0.14 ± 0.06)^33^. Despite the accuracy of predictions being lower in mammals than for humans (mean NSTI = 0.03 ± 0.02), it can still provide useful information of functional predictions for mammalian microbiomes^33^. To explore the variation of predicted metagenomes in winter and spring, we performed LEfSe tests to detect KEGG pathways with significantly different abundances between winter and spring samples. We found that seven KEGG pathways (Cell motility, Glycan Biosynthesis and Metabolism, Signal Transduction, Amino Acid Metabolism, Transport and Catabolism, Endocrine System and Neurodegenerative Diseases) were significantly enriched in winter samples, and six KEGG pathways (Transcription, Enzyme Families, Energy Metabolism, Carbohydrate Metabolism, Cellular Processes and Signaling and Metabolism of Cofactors and Vitamins) were significantly increased in spring samples (LDA > 2, P < 0.05) (Fig. 5).

## Discussion

Wild NHPs have successfully radiated into various seasonal habitats^5^. Anatomical and behavioral strategies are generally considered as the primary ways NHPs adapt to temporal fluctuations of food resources^9^,^16^,^17^,^18^. Having a diverse and responsive gut microbial community may be another important adaptive mechanism. The gut microbial community composition and diversity in our study group strongly shifted between the two seasons. This finding supports H1 that gut microbiome diversity will be significantly different in the two seasons. In highly seasonal ecosystems such as Mt. Huangshan, NHPs are exposed to variable temperatures and rainfall and undergo corresponding seasonal shifts in home range use and food resources. Previous studies show that these factors can affect gut microbiome composition^3^,^34^,^35^. From winter to spring, monkeys in our study group experience a seasonal shift in food type and quality, temperature, and rainfall^29^. These environmental factors are comparatively homogeneous within seasons but vary more markedly between seasons^29^,^30^, resulting in marked variation of gut microbiota between winter and spring in this population. We found that the differences of inter-individual gut community structure were significantly higher in winter, which revealed that inter-individual gut community structure was more different among individuals in winter and more similar in spring. A possible explanation for this finding is that more intense feeding competition exists among individuals during winter (a period of food scarcity), which results in greater individual differences in resource acquisition. With the increase of food availability in spring, the dietary intake of individuals converges on a more similar pattern as feeding competition declines.

What are the potential consequences of the observed seasonal shifts in the wild Tibetan macaque gut microbial community structure? It is widely accepted that a change of gut microbial composition may allow hosts to digest food more efficiently to meet their energy and nutrient demands^21^,^22^,^24^,^27^,^35^. In highly seasonal ecosystems, NHPs are expected to undergo acute energy loss as they adapt to winter weather^36^. A diverse and responsive gut microbial community may be key to the adaptation process for NHPs living in extreme climates. At the phylum level, our study revealed that the relative abundances of Proteobacteria in the monkeys’ gut are significantly higher in winter than in spring. Previous studies in humans, mice and black howler monkeys have demonstrated that Proteobacteria are mainly associated with energy accumulation^21^,^22^,^35^,^37^. Our results indicate that the increase in this phylum during the winter period may help Tibetan macaques cope with cold weather. Recent controlled experiments in mice showed that cold exposure significantly increased the phylum Proteobacteria, and this change helped the host to withstand periods of high energy demand typical during cold periods^35^. We did not detect a significant increase of predicted genes from the metagenome related to energy metabolism pathways in samples collected from our study monkeys during the winter. A possible explanation for this finding is that the food quality of our study group is different from the mice’s diet. From winter to spring, the food resources of Tibetan macaques change from low- to high-quality^29^,^30^. The higher proportion of predicted genes from the metagenome related to energy metabolic pathways might meet the monkeys’ energy metabolism needs in spring.

In addition to cold weather, our study population experiences changes in food quality across seasons. This is problematic for Tibetan macaques during the winter with respect to metabolizing cellulose and dietary fibers found in mature leaves, roots and other vegetative parts that they rely upon at that time of year. A comparison of our winter and spring results show that the most abundant microbiome genus, *Succinivibrio*, significantly increased in winter samples. Species belonging to *Succinivibrio* enrich rumen microbial ecosystems and are efficient in fermenting glucose through the production of acetic and succinic acids^38^, as well as in aiding in the metabolization of different types of fatty acids^39^, which may increase energy utilization efficiency for monkeys during winter.

The genus *Clostridium* contains organisms known to digest cellulose and hemicellulose^40^ and includes many genes that code for cellulose and hemicellulose-digestive enzymes found in the giant panda gut microbiome^41^. The significant increase of *Clostridium sensu stricto* detected in the Tibetan macaque gut microbiome in winter suggests its potential utility for digesting cellulose and dietary fiber. Our PICRUSt analysis also revealed that predicted genes from the metagenome related to glycan biosynthesis and metabolic pathways are significantly increased in winter samples. This pathway is also present at high levels in the wild panda’s gut microbiome^41^ and is beneficial in digesting glycan produced by the breakdown of cellulose and hemicellulose. Thus, we propose that the pattern of gut microbiota found in Tibetan macaques during the winter may increase energy-efficient metabolism of winter foods. In spring, the abundance of the genus *Prevotella* significantly increased. During this time, our study monkeys increase their ingestion of young leaves, which are richer in more easily digestible energy and protein than mature leaves, roots and other vegetative parts^29^,^30^. Species in the genus *Prevotella* are associated with digestion of hemicellulose, pectin, carbohydrate and simple sugars, such as those found in fruits, cereals and young leaves^3^,^42^. The predicted genes from the metagenome related to the Carbohydrate Metabolism and Energy Metabolism pathways were also significantly increased in our spring samples. These results indicate that the changes measured in the gut microbial community in spring may be beneficial for a rapid recovery from acute energy and nutrition loss experienced during the cold winter months.

A recent human intervention study showed that rapid and reproducible changes in microbial community structure and function occur with the consumption of an animal-based versus a plant-based diet^43^. Similar evidence has also been found in wild mice and black howler monkeys^3^,^34^,^44^. These results, considered together with our current findings from wild Tibetan macaques, lead us to speculate that the symbiotic relationship between mammals and their gut microbiota may be an adaptive mechanism for solving dietary dilemmas imposed by seasonal fluctuations in food availability. Further research is necessary to test this hypothesis in more mammals, as well as to better understand the trade-off between nutrition and health via shifts in gut microbiota composition.

## Material and Methods

### Sample collection and Ethics statement

Because the monkeys in our study group are habituated, it is possible to follow them and collect fresh fecal samples. A total of 48 fecal samples were obtained from 33 identified individuals, representing 89.2% of the study group. Among them, 21 different individuals’ feces were sampled over two discrete periods in winter (Jan. 1 to 18, 2015) and 27 individuals in spring (March 24 to April 21, 2015). Sample and individual information are listed in the Supplementary Table S1. All fecal samples were collected, stored and shipped in RNAlater (QIA-GEN, Valencia CA). Samples were shipped at ambient temperatures but subsequently stored at −80 °C. All animal work was approved by the Institutional Animal Care and Use Committee of the Anhui Zoological Society (permit number BH20131202). All experiments were performed in accordance with the approved guidelines and regulations.

### DNA extraction and sequencing

Frozen fecal samples were thawed on ice and dissected. To avoid soil contamination, DNA was then extracted from the inner part of the fecal samples. Total DNA was extracted from frozen stool samples using the QIAampt DNA Stool Mini Kit (Qiagen, Inc., Valencia CA), following the manufacturer’s protocol for pathogen detection, with a modified protocol of the bead-beating procedure described by Schnorr *et al*.^45^. Total DNA extracted from 48 frozen stool samples was sent to the Genome Sequencing of Human Genome Research Centre, Shanghai, China, to carry out sequencing. The V3-V4 regions of 16S rDNA were individually purified with a Min Elute PCR Purification Kit (QIAGEN) and then quantified using the Qubit dsDNA HS assay kit (Invitrogen, American). After the individual quantification step, amplicons were pooled in equal amounts, and pair-end 2 × 200 bp sequencing was performed using the Illlumina Miseq platform and Miseq Reagent Kit v3.

### Sequence analysis

Quality filtering and processing of sequences was performed in QIIME v1.7. The sequences containing the ambiguous base (N) were removed using in-house perl scripts, and only those sequences with an average quality score >25 were included in the analysis. Mothur^46^ was used to determine OTUs and calculate rarefaction curves, rank-abundance plots, Chao1 richness estimates and Shannon diversity estimates according to MiSeq SOP (http://www.mothur.org/wiki/MiSeq_SOP). Sequences were clustered into 97% OTUs through a mothur-formatted version of the RDP training set (v.9) and assigned to respective taxonomic levels (phylum, class, order, family, and genus).

### Data analysis

Beta diversity (between sample diversity) was estimated by computing from the phylogenetic tree the unweighted and weighted UniFrac distances^47^ between samples. Weighted and unweighted UniFrac distances of genus-level relative abundance were used to perform PCoA using the packages Made4 and Vegan (http://www.cran.r-project.org/package=vegan). Multivariate analysis (Permanova test) was used to detect the influence of age, sex and season on beta diversity (unweighted and weighted UniFrac distances) variation, and a Permanova test was performed in the vegan package of R with the function of GUniFrac^48^. Following Maurice, *et al*.^44^, we used linear mixed models to detect the potential multiple influences of season, age and sex on alpha diversity. Each individual’s ID was controlled as a random intercept term. Model assumptions were checked by examining the distribution of residuals and plotting fitted values against residuals; the response variable was log-transformed or square root where needed to meet the model assumptions. The same sets of predictors (age, sex, and season) were included in all starting models. To detect bacterial taxa (including phylum and genus) and KEGG pathways with significantly different abundances between winter and spring samples, LEfSe analysis was used according to the online protocol (https://huttenhower.sph.harvard.edu /galaxy/).

To explore the functional profiles of our bacterial community data set, the functional profiles of microbial communities were predicted using PICRUSt^33^ (Phylogenetic Investigation of Communities by Reconstruction of Unobserved States) according to the online protocol (http://picrust.github.io/picrust/). For the analysis, OTUs were derived from the 16S rRNA gene present in the Greengenes database.

## Additional Information

**Accession codes:** The raw sequences of this study have been deposited in the Sequence Read Archive (accession number SRP073002) .

**How to cite this article**: Sun, B. *et al*. Marked variation between winter and spring gut microbiota in free-ranging Tibetan Macaques (*Macaca thibetana*). *Sci. Rep.* **6**, 26035; doi: 10.1038/srep26035 (2016).

## Supplementary Material

Supplementary Information
