# Supplementary material for: Marked variation between winter and spring gut microbiota in free-ranging Tibetan Macaques (Macaca thibetana)
Source: Sci Rep. 2016 May 16;6:26035. doi: 10.1038/srep26035 (PMC4867428; doi:10.1038/srep26035)
Supplement: Supplementary Information [file srep26035-s1.pdf]

**Supplementary Material**

**"Marked variation between winter and spring gut microbiota in free-ranging Tibetan Macaques (*Macaca thibetana*)"**

Binghua Sun, Xi Wang, Sofi Bernstein, Michael A. Huffman, Dong-Po Xia, Zhiyuan Gu1, Rui Chen, Lori K. Sheeran, R.S. Wagner, Jinhua Li

**Supplementary Table S1**. Summary of sample information and alpha diversity

| Individuals ID | Sex | Age Class | Sampling Period | Sampling Date | OTUs | ACE | Chao | Shannon | Simpson | Coverage |
|---|---|---|---|---|---|---|---|---|---|---|
| YRX-W | Female | Immature | Winter | 2015.01.01 | 989 | 4047 | 2226 | 4.32 | 0.085 | 0.949 |
| TXL-W | Male | Immature | Winter | 2015.01.04 | 1156 | 5155 | 2954 | 5.14 | 0.019 | 0.921 |
| TRY-W | Male | Immature | Winter | 2015.01.05 | 3213 | 23218 | 11045 | 5.61 | 0.021 | 0.853 |
| THY-W | Female | Adult | Winter | 2015.01.11 | 1358 | 6526 | 3477 | 4.39 | 0.066 | 0.942 |
| BT-W | Male | Adult | Winter | 2015.01.11 | 1302 | 7022 | 4264 | 4.57 | 0.05 | 0.933 |
| TH-W | Female | Adult | Winter | 2015.01.11 | 1077 | 4466 | 2725 | 4.53 | 0.054 | 0.943 |
| TXH-W | Female | Adult | Winter | 2015.01.08 | 1362 | 5423 | 3580 | 5.11 | 0.018 | 0.935 |
| TRG-W | Male | Immature | Winter | 2015.01.11 | 804 | 3718 | 2128 | 3.73 | 0.112 | 0.943 |
| TRYx-W | Female | Adult | Winter | 2015.01.08 | 1358 | 6919 | 3871 | 4.85 | 0.039 | 0.933 |
| TR-W | Female | Adult | Winter | 2015.01.10 | 1885 | 10219 | 5448 | 4.52 | 0.065 | 0.931 |
| ZL-W | Male | Adult | Winter | 2015.01.12 | 1114 | 4474 | 2885 | 4.87 | 0.023 | 0.942 |
| HT-W | Male | Adult | Winter | 2015.01.08 | 994 | 3284 | 2186 | 5.26 | 0.013 | 0.938 |
| TXX-W | Female | Adult | Winter | 2015.01.08 | 1254 | 6485 | 4075 | 4.65 | 0.059 | 0.928 |
| YCLO-W | Male | Immature | Winter | 2015.01.04 | 1643 | 7506 | 4658 | 5.18 | 0.024 | 0.936 |
| DS-W | Male | Adult | Winter | 2015.01.12 | 939 | 3850 | 2251 | 4.43 | 0.054 | 0.95 |
| YZ-W | Female | Adult | Winter | 2015.01.05 | 1407 | 6004 | 3590 | 4.8 | 0.031 | 0.954 |
| HH-W | Female | Adult | Winter | 2015.01.11 | 1061 | 4134 | 2498 | 4.1 | 0.089 | 0.952 |
| YXK-W | Male | Immature | Winter | 2015.01.03 | 874 | 3405 | 2099 | 4.62 | 0.033 | 0.938 |
| ZB-W | Male | Adult | Winter | 2015.01.06 | 863 | 3428 | 2270 | 4.9 | 0.022 | 0.924 |
| YM-W | Female | Adult | Winter | 2015.01.12 | 943 | 3545 | 2323 | 4.74 | 0.029 | 0.945 |
| YRB-W | Male | Adult | Winter | 2015.01.18 | 1059 | 6090 | 3270 | 4.65 | 0.046 | 0.92 |
| YRQ-S | Male | Immature | Spring | 2015.03.30 | 1071 | 3409 | 2376 | 4.87 | 0.029 | 0.956 |
| HXM-S | Male | Immature | Spring | 2015.03.31 | 1412 | 5501 | 3954 | 5.16 | 0.022 | 0.944 |
| ZL-S | Male | Adult | Spring | 2015.04.01 | 1197 | 4673 | 2936 | 4.74 | 0.028 | 0.945 |
| YH-S | Female | Adult | Spring | 2015.04.01 | 1371 | 5502 | 3532 | 5.15 | 0.018 | 0.944 |
| YCLA-S | Female | Immature | Spring | 2015.03.31 | 1160 | 3105 | 2253 | 5.17 | 0.02 | 0.957 |
| YRL-S | Female | Immature | Spring | 2015.04.03 | 1338 | 5416 | 3167 | 5.21 | 0.02 | 0.922 |
| ZB-S | Male | Adult | Spring | 2015.03.30 | 1370 | 4351 | 3167 | 5.2 | 0.019 | 0.95 |
| TRY-S | Male | Immature | Spring | 2015.03.24 | 948 | 3315 | 1995 | 4.34 | 0.064 | 0.951 |
| TXX-S | Female | Adult | Spring | 2015.03.30 | 1236 | 5632 | 3352 | 5.11 | 0.023 | 0.924 |
| HT-S | Male | Adult | Spring | 2015.03.30 | 1198 | 4860 | 2948 | 5.3 | 0.017 | 0.931 |

| | | | | | | | | | | |
|---|---|---|---|---|---|---|---|---|---|---|
| TRG-S | Male | Immature | Spring | 2015.04.21 | 1134 | 3887 | 2533 | 4.74 | 0.049 | 0.93 |
| YM-S | Female | Adult | Spring | 2015.04.03 | 1198 | 6593 | 3421 | 4.74 | 0.054 | 0.911 |
| HXW-S | Female | Immature | Spring | 2015.03.30 | 1265 | 5829 | 3721 | 5.35 | 0.015 | 0.929 |
| GS-S | Male | Adult | Spring | 2015.04.21 | 1249 | 8041 | 4075 | 4.3 | 0.072 | 0.948 |
| YZ-S | Female | Adult | Spring | 2015.03.30 | 870 | 3884 | 2145 | 4.21 | 0.046 | 0.957 |
| TT-S | Female | Adult | Spring | 2015.03.30 | 989 | 2751 | 2027 | 5.02 | 0.022 | 0.952 |
| TXH-S | Female | Adult | Spring | 2015.04.10 | 1071 | 3981 | 2404 | 5.3 | 0.011 | 0.939 |
| DS-S | Male | Adult | Spring | 2015.04.14 | 872 | 2344 | 1926 | 4.72 | 0.038 | 0.963 |
| YCY-S | Female | Adult | Spring | 2015.04.13 | 1447 | 6440 | 3814 | 5.19 | 0.022 | 0.919 |
| YXX-S | Female | Adult | Spring | 2015.04.10 | 1381 | 5341 | 3383 | 4.83 | 0.036 | 0.941 |
| TH-S | Female | Adult | Spring | 2015.04.20 | 930 | 3570 | 2245 | 4.92 | 0.025 | 0.927 |
| TG-S | Male | Adult | Spring | 2015.04.04 | 2416 | 15950 | 7644 | 5.47 | 0.019 | 0.917 |
| HH-S | Female | Adult | Spring | 2015.04.12 | 1053 | 3967 | 2516 | 4.81 | 0.044 | 0.934 |
| YRB-S | Male | Adult | Spring | 2015.04.20 | 1331 | 6600 | 3742 | 4.99 | 0.03 | 0.921 |
| YXK-S | Male | Immature | Spring | 2015.04.10 | 1369 | 6732 | 3685 | 5.18 | 0.019 | 0.935 |
| BT-S | Male | Adult | Spring | 2015.04.01 | 754 | 3041 | 1817 | 4.19 | 0.09 | 0.943 |
| JT-S | Male | Adult | Spring | 2015.04.10 | 1294 | 6135 | 3234 | 5.2 | 0.025 | 0.918 |

**Supplementary Table S2**. Taxonomy of Tibetan macaques' gut microbiome at phylum level (the mean relative abundance of per sample > 0.01% was presented, each number in the table cell represent the proportion)

| Samples ID | Sampling period | Taxon （phylum） | | | | | |
|---|---|---|---|---|---|---|---|
| | | Spirochaetia | Proteobacteria | Firmicutes | Bacteroidetes | Actinobacteria | Verrucomicrobia |
| YRX-W | winter | 0.01406 | 0.30646 | 0.39828 | 0.24926 | 0.00885 | 0.00000 |
| TXL-W | winter | 0.01505 | 0.10832 | 0.38449 | 0.46216 | 0.00022 | 0.00055 |
| TRY-W | winter | 0.08173 | 0.09133 | 0.42662 | 0.34729 | 0.00191 | 0.00055 |
| THY-W | winter | 0.03754 | 0.25813 | 0.30471 | 0.36960 | 0.01629 | 0.00007 |
| BT-W | winter | 0.02456 | 0.15715 | 0.42053 | 0.33714 | 0.00180 | 0.00086 |
| TH-W | winter | 0.00548 | 0.24441 | 0.38798 | 0.33003 | 0.00966 | 0.00096 |
| TXH-W | winter | 0.06829 | 0.06659 | 0.43434 | 0.40543 | 0.00070 | 0.00101 |
| TRG-W | winter | 0.00882 | 0.32877 | 0.22608 | 0.25266 | 0.00226 | 0.00068 |
| TRYx-W | winter | 0.01712 | 0.21671 | 0.37944 | 0.31462 | 0.00085 | 0.00108 |
| TR-W | winter | 0.08237 | 0.24893 | 0.28634 | 0.35201 | 0.01273 | 0.00011 |
| ZL-W | winter | 0.00545 | 0.07610 | 0.50686 | 0.35177 | 0.00767 | 0.00281 |
| HT-W | winter | 0.00739 | 0.07073 | 0.49737 | 0.34191 | 0.00045 | 0.00134 |
| TXX-W | winter | 0.00400 | 0.22100 | 0.36501 | 0.34074 | 0.00053 | 0.00053 |
| YCLO-W | winter | 0.01553 | 0.11027 | 0.38448 | 0.43402 | 0.00069 | 0.00119 |
| DS-W | winter | 0.01186 | 0.23954 | 0.28772 | 0.37276 | 0.00552 | 0.00091 |
| YZ-W | winter | 0.01207 | 0.14978 | 0.47836 | 0.31749 | 0.00060 | 0.00011 |
| HH-W | winter | 0.06387 | 0.38781 | 0.31683 | 0.19289 | 0.02117 | 0.00000 |
| YXK-W | winter | 0.02329 | 0.11074 | 0.37844 | 0.41453 | 0.00058 | 0.00210 |
| ZB-W | winter | 0.04724 | 0.09882 | 0.43369 | 0.39281 | 0.00188 | 0.00116 |
| YM-W | winter | 0.00523 | 0.16198 | 0.44247 | 0.31785 | 0.00177 | 0.00276 |
| YRB-W | winter | 0.04560 | 0.21377 | 0.36579 | 0.35021 | 0.00321 | 0.00057 |
| YRQ-S | spring | 0.00267 | 0.05353 | 0.56698 | 0.34556 | 0.00114 | 0.00183 |

| | | | | | | | |
|---|---|---|---|---|---|---|---|
| HXM-S | spring | 0.00347 | 0.10427 | 0.52608 | 0.33142 | 0.00193 | 0.00047 |
| ZL-S | spring | 0.00047 | 0.04904 | 0.59866 | 0.33512 | 0.00179 | 0.00000 |
| YH-S | spring | 0.00680 | 0.11929 | 0.49678 | 0.35976 | 0.00287 | 0.00028 |
| YCLA-S | spring | 0.00045 | 0.05205 | 0.57893 | 0.32811 | 0.00327 | 0.00007 |
| YRL-S | spring | 0.05118 | 0.05811 | 0.42496 | 0.41861 | 0.00183 | 0.00096 |
| ZB-S | spring | 0.01672 | 0.05233 | 0.54348 | 0.35824 | 0.00303 | 0.00092 |
| TRY-S | spring | 0.01748 | 0.23828 | 0.42367 | 0.29263 | 0.01667 | 0.00018 |
| TXX-S | spring | 0.00866 | 0.06699 | 0.48188 | 0.41569 | 0.00119 | 0.00109 |
| HT-S | spring | 0.00952 | 0.02277 | 0.60149 | 0.31881 | 0.00461 | 0.00128 |
| TRG-S | spring | 0.00637 | 0.18771 | 0.40057 | 0.36448 | 0.00191 | 0.00138 |
| YM-S | spring | 0.00755 | 0.17549 | 0.35762 | 0.40934 | 0.00526 | 0.00275 |
| HXW-S | spring | 0.01239 | 0.13106 | 0.55818 | 0.26730 | 0.00666 | 0.00037 |
| GS-S | spring | 0.06178 | 0.27426 | 0.37309 | 0.23284 | 0.01769 | 0.00019 |
| YZ-S | spring | 0.00098 | 0.17896 | 0.37369 | 0.40556 | 0.02927 | 0.00000 |
| TT-S | spring | 0.00622 | 0.15031 | 0.50801 | 0.30580 | 0.00254 | 0.00188 |
| TXH-S | spring | 0.00369 | 0.06396 | 0.52634 | 0.36460 | 0.00190 | 0.00170 |
| DS-S | spring | 0.02189 | 0.05036 | 0.49054 | 0.28486 | 0.00245 | 0.00237 |
| YCY-S | spring | 0.00431 | 0.09375 | 0.42245 | 0.44281 | 0.00153 | 0.00197 |
| YXX-S | spring | 0.00370 | 0.15862 | 0.40053 | 0.40942 | 0.00398 | 0.00014 |
| TH-S | spring | 0.01889 | 0.08425 | 0.41039 | 0.45995 | 0.01098 | 0.00107 |
| TG-S | spring | 0.01849 | 0.06806 | 0.48818 | 0.40116 | 0.00177 | 0.00162 |
| HH-S | spring | 0.01263 | 0.05393 | 0.44347 | 0.43720 | 0.00127 | 0.00138 |
| YRB-S | spring | 0.00232 | 0.12336 | 0.40418 | 0.45267 | 0.00149 | 0.00065 |
| YXK-S | spring | 0.00415 | 0.11236 | 0.45158 | 0.40017 | 0.00177 | 0.00184 |
| BT-S | spring | 0.06300 | 0.30787 | 0.36245 | 0.24461 | 0.00115 | 0.00051 |
| JT-S | spring | 0.01170 | 0.14183 | 0.52823 | 0.26381 | 0.00224 | 0.00051 |

**Supplementary Table S3.** Taxonomy of Tibetan macaques' gut microbiome at genus level (the mean relative abundance of per sample > 0.01% was presented, each number in the table cell represent the proportion)

| Taxon （genus） | Samples ID | | | | | | | | | | | |
|---|---|---|---|---|---|---|---|---|---|---|---|---|
| | YRQ-S | HXM-S | ZL-S | YH-S | YCLA-S | YRL-S | ZB-S | TRY-S | TXX-S | HT-S | TRG-S | YM-S |
| *Succinivibrio* | 0.2435 | 0.2015 | 0.2652 | 0.1946 | 0.2134 | 0.2081 | 0.1729 | 0.2326 | 0.2883 | 0.2355 | 0.2329 | 0.1286 |
| *Prevotella* | 0.0114 | 0.0822 | 0.0250 | 0.0209 | 0.0045 | 0.0399 | 0.0315 | 0.2258 | 0.0310 | 0.0016 | 0.1762 | 0.1439 |
| *Porphyromonadaceae (OTU00002)* | 0.0464 | 0.0206 | 0.0585 | 0.0175 | 0.0262 | 0.0308 | 0.0439 | 0.0596 | 0.0512 | 0.0977 | 0.0333 | 0.0333 |
| *Treponema* | 0.0049 | 0.0078 | 0.0125 | 0.0025 | 0.0004 | 0.0826 | 0.0120 | 0.0030 | 0.0455 | 0.0000 | 0.0260 | 0.1733 |
| *Helicobacter* | 0.0255 | 0.0091 | 0.0099 | 0.0643 | 0.0367 | 0.0038 | 0.0124 | 0.0038 | 0.0125 | 0.0151 | 0.0030 | 0.0112 |
| *Bacteroidetes (OTU00009)* | 0.0700 | 0.0199 | 0.0103 | 0.0084 | 0.0125 | 0.0130 | 0.0317 | 0.0063 | 0.0120 | 0.0186 | 0.0124 | 0.0094 |
| *Faecalibacterium* | 0.0280 | 0.0141 | 0.0032 | 0.0370 | 0.0221 | 0.0121 | 0.0149 | 0.0089 | 0.0084 | 0.0124 | 0.0131 | 0.0118 |
| *Oscillibacter* | 0.0027 | 0.0035 | 0.0005 | 0.0068 | 0.0004 | 0.0512 | 0.0167 | 0.0175 | 0.0087 | 0.0095 | 0.0064 | 0.0076 |
| *Clostridium XlVa* | 0.0042 | 0.0083 | 0.0045 | 0.0176 | 0.0053 | 0.0225 | 0.0046 | 0.0523 | 0.0119 | 0.0027 | 0.0116 | 0.0074 |
| *Ruminococcus* | 0.0170 | 0.0034 | 0.0418 | 0.0228 | 0.0092 | 0.0059 | 0.0095 | 0.0090 | 0.0227 | 0.0196 | 0.0027 | 0.0048 |
| *Clostridium sensu stricto* | 0.0326 | 0.0012 | 0.0001 | 0.0386 | 0.0040 | 0.0183 | 0.0264 | 0.0029 | 0.0313 | 0.0077 | 0.0197 | 0.0064 |
| *Alloprevotella* | 0.0145 | 0.0108 | 0.0189 | 0.0119 | 0.0080 | 0.0076 | 0.0085 | 0.0086 | 0.0204 | 0.0106 | 0.0044 | 0.0043 |
| *Dialister* | 0.0005 | 0.0055 | 0.0063 | 0.0099 | 0.0261 | 0.0022 | 0.0091 | 0.0280 | 0.0008 | 0.0126 | 0.0121 | 0.0016 |
| *Barnesiella* | 0.0078 | 0.0047 | 0.0272 | 0.0044 | 0.0110 | 0.0072 | 0.0029 | 0.0064 | 0.0061 | 0.0069 | 0.0075 | 0.0300 |
| *Clostridium XI* | 0.0013 | 0.0034 | 0.0300 | 0.0102 | 0.0046 | 0.0047 | 0.0038 | 0.0112 | 0.0066 | 0.0050 | 0.0022 | 0.0042 |
| *Blautia* | 0.0069 | 0.0059 | 0.0033 | 0.0023 | 0.0067 | 0.0087 | 0.0030 | 0.0017 | 0.0101 | 0.0187 | 0.0019 | 0.0157 |

| | | | | | | | | | | | | |
|---|---|---|---|---|---|---|---|---|---|---|---|---|
| *Roseburia* | 0.0066 | 0.0059 | 0.0085 | 0.0070 | 0.0102 | 0.0031 | 0.0074 | 0.0094 | 0.0049 | 0.0169 | 0.0039 | 0.0048 |
| *Coprococcus* | 0.0008 | 0.0050 | 0.0003 | 0.0043 | 0.0004 | 0.0036 | 0.0099 | 0.0197 | 0.0020 | 0.0002 | 0.0049 | 0.0056 |
| *Lachnospiracea_ incertae_sedis* | 0.0008 | 0.0033 | 0.0000 | 0.0022 | 0.0034 | 0.0184 | 0.0005 | 0.0149 | 0.0075 | 0.0003 | 0.0075 | 0.0074 |
| *Escherichia/Shigella* | 0.0027 | 0.0015 | 0.0091 | 0.0060 | 0.0036 | 0.0018 | 0.0053 | 0.0092 | 0.0057 | 0.0094 | 0.0013 | 0.0019 |
| *Clostridium IV* | 0.0088 | 0.0022 | 0.0002 | 0.0107 | 0.0044 | 0.0023 | 0.0084 | 0.0038 | 0.0054 | 0.0048 | 0.0030 | 0.0062 |
| *Butyricicoccus* | 0.0023 | 0.0059 | 0.0006 | 0.0008 | 0.0027 | 0.0013 | 0.0049 | 0.0050 | 0.0193 | 0.0081 | 0.0046 | 0.0014 |
| *Catenibacterium* | 0.0005 | 0.0041 | 0.0068 | 0.0025 | 0.0032 | 0.0048 | 0.0024 | 0.0022 | 0.0057 | 0.0034 | 0.0047 | 0.0064 |
| *Anaerostipes* | 0.0084 | 0.0037 | 0.0010 | 0.0114 | 0.0042 | 0.0015 | 0.0040 | 0.0055 | 0.0042 | 0.0028 | 0.0003 | 0.0008 |
| *Gemmiger* | 0.0051 | 0.0030 | 0.0012 | 0.0050 | 0.0040 | 0.0019 | 0.0051 | 0.0009 | 0.0032 | 0.0047 | 0.0015 | 0.0034 |
| *Subdivision5_genera_ incertae_sedis* | 0.0008 | 0.0009 | 0.0017 | 0.0048 | 0.0021 | 0.0014 | 0.0011 | 0.0029 | 0.0017 | 0.0014 | 0.0012 | 0.0011 |
| *Flavonifractor* | 0.0011 | 0.0027 | 0.0028 | 0.0015 | 0.0025 | 0.0008 | 0.0016 | 0.0006 | 0.0013 | 0.0009 | 0.0023 | 0.0003 |
| *Dorea* | 0.0008 | 0.0018 | 0.0043 | 0.0013 | 0.0008 | 0.0010 | 0.0016 | 0.0002 | 0.0011 | 0.0017 | 0.0010 | 0.0001 |
| *Clostridium XlVb* | 0.0018 | 0.0005 | 0.0000 | 0.0003 | 0.0001 | 0.0010 | 0.0009 | 0.0002 | 0.0011 | 0.0013 | 0.0014 | 0.0026 |
| *Erysipelotrichaceae_ incertae_sedis* | 0.0000 | 0.0002 | 0.0003 | 0.0009 | 0.0005 | 0.0002 | 0.0003 | 0.0005 | 0.0005 | 0.0009 | 0.0003 | 0.0001 |

| Taxon （genus） | Samples ID | | | | | | | | | | | |
|---|---|---|---|---|---|---|---|---|---|---|---|---|
| | HXW-S | GS-S | YZ-S | TT-S | TXH-S | DS-S | YCY-S | YXX-S | TH-S | TG-S | HH-S | YRB-S |
| *Succinivibrio* | 0.1861 | 0.0978 | 0.3781 | 0.2075 | 0.1298 | 0.0353 | 0.2515 | 0.2993 | 0.3500 | 0.2516 | 0.1288 | 0.3389 |
| *Prevotella* | 0.0470 | 0.2539 | 0.1348 | 0.0623 | 0.0173 | 0.0018 | 0.0718 | 0.1230 | 0.0648 | 0.0417 | 0.0235 | 0.0806 |
| *Porphyromonadaceae (OTU00002)* | 0.0271 | 0.0054 | 0.0311 | 0.0390 | 0.0343 | 0.0065 | 0.0458 | 0.0711 | 0.0954 | 0.0401 | 0.0278 | 0.0287 |
| *Treponema* | 0.0134 | 0.0295 | 0.0000 | 0.0073 | 0.0190 | 0.0237 | 0.0494 | 0.0267 | 0.0063 | 0.0435 | 0.1884 | 0.0066 |
| *Helicobacter* | 0.0733 | 0.0081 | 0.0252 | 0.0731 | 0.0046 | 0.0056 | 0.0048 | 0.0095 | 0.0050 | 0.0096 | 0.0177 | 0.0120 |
| *Bacteroidetes (OTU00009)* | 0.0200 | 0.0014 | 0.0098 | 0.0138 | 0.0125 | 0.0128 | 0.0376 | 0.0069 | 0.0095 | 0.0098 | 0.0157 | 0.0158 |
| *Faecalibacterium* | 0.0158 | 0.0196 | 0.0025 | 0.0135 | 0.0278 | 0.0226 | 0.0175 | 0.0156 | 0.0091 | 0.0103 | 0.0091 | 0.0120 |
| *Oscillibacter* | 0.0124 | 0.0617 | 0.0010 | 0.0062 | 0.0037 | 0.0219 | 0.0043 | 0.0036 | 0.0189 | 0.0185 | 0.0126 | 0.0023 |
| *Clostridium XlVa* | 0.0070 | 0.0217 | 0.0333 | 0.0039 | 0.0092 | 0.0014 | 0.0095 | 0.0193 | 0.0344 | 0.0135 | 0.0032 | 0.0203 |
| *Ruminococcus* | 0.0088 | 0.0028 | 0.0713 | 0.0120 | 0.0032 | 0.0035 | 0.0048 | 0.0068 | 0.0100 | 0.0086 | 0.0065 | 0.0094 |
| *Clostridium sensu stricto* | 0.0172 | 0.0114 | 0.0004 | 0.0044 | 0.0213 | 0.0000 | 0.0153 | 0.0096 | 0.0054 | 0.0106 | 0.0006 | 0.0080 |
| *Alloprevotella* | 0.0154 | 0.0038 | 0.0183 | 0.0151 | 0.0080 | 0.0047 | 0.0026 | 0.0051 | 0.0151 | 0.0065 | 0.0072 | 0.0135 |
| *Dialister* | 0.0006 | 0.0110 | 0.0083 | 0.0214 | 0.0171 | 0.0000 | 0.0137 | 0.0018 | 0.0142 | 0.0020 | 0.0029 | 0.0145 |
| *Barnesiella* | 0.0056 | 0.0040 | 0.0037 | 0.0059 | 0.0054 | 0.0003 | 0.0085 | 0.0063 | 0.0283 | 0.0084 | 0.0023 | 0.0159 |
| *Clostridium XI* | 0.0086 | 0.0126 | 0.0134 | 0.0020 | 0.0031 | 0.0004 | 0.0035 | 0.0056 | 0.0046 | 0.0230 | 0.0050 | 0.0265 |
| *Blautia* | 0.0096 | 0.0002 | 0.0046 | 0.0088 | 0.0061 | 0.0128 | 0.0051 | 0.0055 | 0.0038 | 0.0191 | 0.0205 | 0.0038 |
| *Roseburia* | 0.0059 | 0.0010 | 0.0154 | 0.0089 | 0.0068 | 0.0014 | 0.0033 | 0.0094 | 0.0023 | 0.0157 | 0.0040 | 0.0067 |
| *Coprococcus* | 0.0013 | 0.0150 | 0.0069 | 0.0013 | 0.0010 | 0.0001 | 0.0011 | 0.0009 | 0.0048 | 0.0186 | 0.0005 | 0.0034 |
| *Lachnospiracea_ incertae_sedis* | 0.0022 | 0.0013 | 0.0008 | 0.0057 | 0.0083 | 0.0000 | 0.0046 | 0.0075 | 0.0060 | 0.0002 | 0.0039 | 0.0005 |
| *Escherichia/Shigella* | 0.0059 | 0.0012 | 0.0033 | 0.0073 | 0.0034 | 0.0016 | 0.0025 | 0.0026 | 0.0095 | 0.0055 | 0.0013 | 0.0059 |
| *Clostridium IV* | 0.0031 | 0.0002 | 0.0033 | 0.0047 | 0.0005 | 0.0034 | 0.0019 | 0.0016 | 0.0028 | 0.0104 | 0.0006 | 0.0046 |
| *Butyricicoccus* | 0.0029 | 0.0003 | 0.0015 | 0.0110 | 0.0054 | 0.0003 | 0.0046 | 0.0020 | 0.0007 | 0.0028 | 0.0024 | 0.0014 |
| *Catenibacterium* | 0.0028 | 0.0003 | 0.0026 | 0.0024 | 0.0049 | 0.0008 | 0.0042 | 0.0075 | 0.0027 | 0.0066 | 0.0051 | 0.0049 |
| *Anaerostipes* | 0.0017 | 0.0014 | 0.0162 | 0.0030 | 0.0016 | 0.0074 | 0.0006 | 0.0014 | 0.0021 | 0.0006 | 0.0021 | 0.0007 |
| *Gemmiger* | 0.0033 | 0.0003 | 0.0010 | 0.0026 | 0.0049 | 0.0035 | 0.0022 | 0.0021 | 0.0039 | 0.0029 | 0.0035 | 0.0026 |
| *Subdivision5_genera_ incertae_sedis* | 0.0023 | 0.0009 | 0.0102 | 0.0017 | 0.0015 | 0.0000 | 0.0015 | 0.0033 | 0.0043 | 0.0060 | 0.0012 | 0.0073 |
| *Flavonifractor* | 0.0009 | 0.0026 | 0.0002 | 0.0023 | 0.0012 | 0.0012 | 0.0012 | 0.0014 | 0.0008 | 0.0021 | 0.0003 | 0.0017 |
| *Dorea* | 0.0010 | 0.0002 | 0.0002 | 0.0003 | 0.0026 | 0.0004 | 0.0006 | 0.0011 | 0.0025 | 0.0005 | 0.0011 | 0.0000 |
| *Clostridium XlVb* | 0.0004 | 0.0002 | 0.0000 | 0.0019 | 0.0016 | 0.0024 | 0.0020 | 0.0001 | 0.0011 | 0.0016 | 0.0014 | 0.0007 |
| *Erysipelotrichaceae_* | 0.0003 | 0.0001 | 0.0020 | 0.0003 | 0.0003 | 0.0004 | 0.0005 | 0.0008 | 0.0007 | 0.0005 | 0.0001 | 0.0008 |

*incertae_sedis*

| Taxon （genus） | Samples ID | | | | | | | | | | | |
|---|---|---|---|---|---|---|---|---|---|---|---|---|
| | YXK-S | BT-S | JT-S | YRX-W | TXL-W | TRY-W | THY-W | BT-W | TH-W | TXH-W | TRG-W | TRYx-W |
| *Succinivibrio* | 0.2325 | 0.1106 | 0.1578 | 0.2849 | 0.0538 | 0.0549 | 0.2379 | 0.1242 | 0.2065 | 0.0455 | 0.2827 | 0.1506 |
| *Prevotella* | 0.0835 | 0.2909 | 0.1194 | 0.1817 | 0.1933 | 0.0720 | 0.2873 | 0.0357 | 0.1237 | 0.2022 | 0.1580 | 0.0796 |
| *Porphyromonadaceae (OTU00002)* | 0.0305 | 0.0008 | 0.0781 | 0.0029 | 0.0138 | 0.0701 | 0.0012 | 0.1683 | 0.0839 | 0.0295 | 0.0069 | 0.1053 |
| *Treponema* | 0.0559 | 0.0074 | 0.0001 | 0.0141 | 0.0150 | 0.0817 | 0.0375 | 0.0243 | 0.0055 | 0.0683 | 0.0088 | 0.0170 |
| *Helicobacter* | 0.0025 | 0.0088 | 0.0077 | 0.0107 | 0.0095 | 0.0054 | 0.0028 | 0.0181 | 0.0277 | 0.0060 | 0.0399 | 0.0458 |
| *Bacteroidetes (OTU00009)* | 0.0116 | 0.0078 | 0.0285 | 0.0042 | 0.0269 | 0.0559 | 0.0026 | 0.0358 | 0.0107 | 0.0206 | 0.0024 | 0.0294 |
| *Faecalibacterium* | 0.0199 | 0.0075 | 0.0105 | 0.0338 | 0.0143 | 0.0049 | 0.0447 | 0.0041 | 0.0103 | 0.0322 | 0.0122 | 0.0130 |
| *Oscillibacter* | 0.0042 | 0.0629 | 0.0117 | 0.0132 | 0.0194 | 0.0139 | 0.0156 | 0.0184 | 0.0120 | 0.0149 | 0.0069 | 0.0077 |
| *Clostridium XlVa* | 0.0153 | 0.0110 | 0.0003 | 0.0070 | 0.0128 | 0.0075 | 0.0067 | 0.0160 | 0.0111 | 0.0277 | 0.0137 | 0.0153 |
| *Ruminococcus* | 0.0076 | 0.0027 | 0.0082 | 0.0093 | 0.0100 | 0.0514 | 0.0082 | 0.0058 | 0.0452 | 0.0487 | 0.0135 | 0.0057 |
| *Clostridium sensu stricto* | 0.0199 | 0.0000 | 0.0007 | 0.0687 | 0.0018 | 0.0148 | 0.0426 | 0.0032 | 0.0036 | 0.0096 | 0.0080 | 0.0050 |
| *Alloprevotella* | 0.0089 | 0.0064 | 0.0096 | 0.0062 | 0.0835 | 0.0022 | 0.0200 | 0.0026 | 0.0044 | 0.0146 | 0.0035 | 0.0046 |
| *Dialister* | 0.0111 | 0.0057 | 0.0102 | 0.0161 | 0.0030 | 0.0032 | 0.0167 | 0.0078 | 0.0146 | 0.0047 | 0.0089 | 0.0064 |
| *Barnesiella* | 0.0073 | 0.0038 | 0.0087 | 0.0046 | 0.0037 | 0.0023 | 0.0066 | 0.0015 | 0.0014 | 0.0249 | 0.0055 | 0.0025 |
| *Clostridium XI* | 0.0095 | 0.0045 | 0.0043 | 0.0042 | 0.0042 | 0.0026 | 0.0019 | 0.0015 | 0.0017 | 0.0140 | 0.0028 | 0.0033 |
| *Blautia* | 0.0063 | 0.0001 | 0.0022 | 0.0020 | 0.0046 | 0.0023 | 0.0015 | 0.0014 | 0.0019 | 0.0024 | 0.0029 | 0.0024 |
| *Roseburia* | 0.0046 | 0.0046 | 0.0082 | 0.0010 | 0.0054 | 0.0036 | 0.0036 | 0.0128 | 0.0005 | 0.0022 | 0.0025 | 0.0003 |
| *Coprococcus* | 0.0048 | 0.0253 | 0.0154 | 0.0050 | 0.0018 | 0.0002 | 0.0088 | 0.0013 | 0.0008 | 0.0008 | 0.0012 | 0.0013 |
| *Lachnospiracea_ incertae_sedis* | 0.0104 | 0.0020 | 0.0017 | 0.0014 | 0.0066 | 0.0008 | 0.0023 | 0.0006 | 0.0010 | 0.0028 | 0.0017 | 0.0044 |
| *Escherichia/Shigella* | 0.0050 | 0.0028 | 0.0050 | 0.0013 | 0.0017 | 0.0007 | 0.0015 | 0.0022 | 0.0004 | 0.0032 | 0.0008 | 0.0066 |
| *Clostridium IV* | 0.0020 | 0.0031 | 0.0141 | 0.0036 | 0.0034 | 0.0029 | 0.0009 | 0.0013 | 0.0017 | 0.0026 | 0.0010 | 0.0029 |
| *Butyricicoccus* | 0.0039 | 0.0026 | 0.0032 | 0.0001 | 0.0074 | 0.0005 | 0.0001 | 0.0000 | 0.0001 | 0.0033 | 0.0005 | 0.0074 |
| *Catenibacterium* | 0.0032 | 0.0014 | 0.0026 | 0.0010 | 0.0075 | 0.0014 | 0.0003 | 0.0001 | 0.0004 | 0.0016 | 0.0009 | 0.0063 |
| *Anaerostipes* | 0.0012 | 0.0006 | 0.0004 | 0.0000 | 0.0015 | 0.0007 | 0.0007 | 0.0002 | 0.0001 | 0.0044 | 0.0011 | 0.0040 |
| *Gemmiger* | 0.0025 | 0.0010 | 0.0039 | 0.0022 | 0.0007 | 0.0006 | 0.0030 | 0.0004 | 0.0006 | 0.0015 | 0.0000 | 0.0004 |
| *Subdivision5_genera_ incertae_sedis* | 0.0020 | 0.0011 | 0.0023 | 0.0000 | 0.0006 | 0.0006 | 0.0001 | 0.0009 | 0.0010 | 0.0010 | 0.0007 | 0.0011 |
| *Flavonifractor* | 0.0009 | 0.0022 | 0.0012 | 0.0016 | 0.0007 | 0.0008 | 0.0009 | 0.0010 | 0.0005 | 0.0006 | 0.0005 | 0.0009 |
| *Dorea* | 0.0002 | 0.0001 | 0.0008 | 0.0004 | 0.0017 | 0.0013 | 0.0004 | 0.0002 | 0.0000 | 0.0009 | 0.0006 | 0.0006 |
| *Clostridium XlVb* | 0.0018 | 0.0005 | 0.0005 | 0.0016 | 0.0015 | 0.0001 | 0.0004 | 0.0001 | 0.0006 | 0.0006 | 0.0003 | 0.0002 |
| *Erysipelotrichaceae_ incertae_sedis* | 0.0005 | 0.0000 | 0.0002 | 0.0004 | 0.0001 | 0.0002 | 0.0002 | 0.0001 | 0.0001 | 0.0002 | 0.0003 | 0.0001 |

| Taxon （genus） | Samples ID | | | | | | | | | | | |
|---|---|---|---|---|---|---|---|---|---|---|---|---|
| | HT-W | TXX-W | YCLO-W | DS-W | YZ-W | HH-W | YXK-W | ZB-W | YM-W | YRB-W | TR-W | ZL-W |
| *Succinivibrio* | 0.0383 | 0.1814 | 0.0774 | 0.1975 | 0.0989 | 0.2752 | 0.0629 | 0.0870 | 0.1097 | 0.1815 | 0.2205 | 0.0223 |
| *Prevotella* | 0.1189 | 0.0793 | 0.1450 | 0.2313 | 0.0476 | 0.1147 | 0.0352 | 0.1292 | 0.0704 | 0.1508 | 0.2679 | 0.0580 |
| *Porphyromonadaceae (OTU00002)* | 0.0572 | 0.1556 | 0.0984 | 0.0110 | 0.1094 | 0.0002 | 0.1309 | 0.0316 | 0.0894 | 0.0770 | 0.0037 | 0.0988 |
| *Treponema* | 0.0074 | 0.0040 | 0.0155 | 0.0119 | 0.0121 | 0.0638 | 0.0233 | 0.0472 | 0.0052 | 0.0456 | 0.0823 | 0.0055 |
| *Helicobacter* | 0.0104 | 0.0192 | 0.0201 | 0.0277 | 0.0352 | 0.1026 | 0.0082 | 0.0038 | 0.0336 | 0.0102 | 0.0184 | 0.0316 |
| *Bacteroidetes (OTU00009)* | 0.0346 | 0.0055 | 0.0120 | 0.0364 | 0.0222 | 0.0132 | 0.0348 | 0.0175 | 0.0035 | 0.0308 | 0.0088 | 0.0099 |
| *Faecalibacterium* | 0.0040 | 0.0089 | 0.0155 | 0.0133 | 0.0055 | 0.0374 | 0.0014 | 0.0051 | 0.0090 | 0.0285 | 0.0417 | 0.0029 |
| *Oscillibacter* | 0.0188 | 0.0071 | 0.0187 | 0.0131 | 0.0121 | 0.0154 | 0.0084 | 0.0261 | 0.0292 | 0.0074 | 0.0074 | 0.0294 |
| *Clostridium XlVa* | 0.0339 | 0.0100 | 0.0206 | 0.0155 | 0.0375 | 0.0044 | 0.0235 | 0.0116 | 0.0124 | 0.0095 | 0.0087 | 0.0078 |
| *Ruminococcus* | 0.0079 | 0.0068 | 0.0074 | 0.0082 | 0.0140 | 0.0104 | 0.0064 | 0.0051 | 0.0041 | 0.0149 | 0.0164 | 0.0072 |
| *Clostridium sensu stricto* | 0.0020 | 0.0040 | 0.0031 | 0.0204 | 0.0007 | 0.0293 | 0.0047 | 0.0157 | 0.0047 | 0.0189 | 0.0134 | 0.0026 |

| | | | | | | | | | | | | |
|---|---|---|---|---|---|---|---|---|---|---|---|---|
| *Alloprevotella* | 0.0011 | 0.0113 | 0.0267 | 0.0032 | 0.0088 | 0.0019 | 0.0017 | 0.0004 | 0.0007 | 0.0039 | 0.0073 | 0.0009 |
| *Dialister* | 0.0018 | 0.0085 | 0.0033 | 0.0048 | 0.0078 | 0.0184 | 0.0031 | 0.0052 | 0.0096 | 0.0150 | 0.0132 | 0.0091 |
| *Barnesiella* | 0.0104 | 0.0012 | 0.0073 | 0.0016 | 0.0001 | 0.0020 | 0.0010 | 0.0428 | 0.0003 | 0.0029 | 0.0031 | 0.0001 |
| *Clostridium XI* | 0.0103 | 0.0037 | 0.0092 | 0.0003 | 0.0015 | 0.0033 | 0.0033 | 0.0074 | 0.0023 | 0.0071 | 0.0031 | 0.0003 |
| *Blautia* | 0.0028 | 0.0055 | 0.0064 | 0.0005 | 0.0010 | 0.0081 | 0.0003 | 0.0020 | 0.0009 | 0.0079 | 0.0032 | 0.0012 |
| *Roseburia* | 0.0010 | 0.0025 | 0.0015 | 0.0005 | 0.0007 | 0.0055 | 0.0014 | 0.0027 | 0.0032 | 0.0023 | 0.0045 | 0.0003 |
| *Coprococcus* | 0.0003 | 0.0013 | 0.0015 | 0.0014 | 0.0009 | 0.0152 | 0.0010 | 0.0012 | 0.0003 | 0.0010 | 0.0108 | 0.0000 |
| *Lachnospiracea_ incertae_sedis* | 0.0030 | 0.0042 | 0.0050 | 0.0016 | 0.0023 | 0.0030 | 0.0012 | 0.0004 | 0.0009 | 0.0050 | 0.0044 | 0.0007 |
| *Escherichia/Shigella* | 0.0015 | 0.0009 | 0.0011 | 0.0022 | 0.0065 | 0.0031 | 0.0013 | 0.0030 | 0.0017 | 0.0009 | 0.0006 | 0.0008 |
| *Clostridium IV* | 0.0018 | 0.0025 | 0.0008 | 0.0045 | 0.0011 | 0.0010 | 0.0001 | 0.0017 | 0.0013 | 0.0009 | 0.0015 | 0.0013 |
| *Butyricicoccus* | 0.0027 | 0.0023 | 0.0043 | 0.0004 | 0.0030 | 0.0010 | 0.0006 | 0.0001 | 0.0005 | 0.0015 | 0.0009 | 0.0000 |
| *Catenibacterium* | 0.0016 | 0.0014 | 0.0015 | 0.0001 | 0.0024 | 0.0031 | 0.0001 | 0.0007 | 0.0004 | 0.0032 | 0.0006 | 0.0000 |
| *Anaerostipes* | 0.0006 | 0.0031 | 0.0013 | 0.0008 | 0.0004 | 0.0022 | 0.0016 | 0.0009 | 0.0000 | 0.0027 | 0.0009 | 0.0000 |
| *Gemmiger* | 0.0001 | 0.0007 | 0.0019 | 0.0003 | 0.0007 | 0.0036 | 0.0007 | 0.0007 | 0.0002 | 0.0013 | 0.0044 | 0.0005 |
| *Subdivision5_genera_ incertae_sedis* | 0.0013 | 0.0005 | 0.0011 | 0.0009 | 0.0001 | 0.0000 | 0.0021 | 0.0012 | 0.0028 | 0.0006 | 0.0001 | 0.0028 |
| *Flavonifractor* | 0.0007 | 0.0005 | 0.0010 | 0.0005 | 0.0011 | 0.0007 | 0.0001 | 0.0009 | 0.0008 | 0.0009 | 0.0007 | 0.0009 |
| *Dorea* | 0.0002 | 0.0005 | 0.0016 | 0.0001 | 0.0006 | 0.0006 | 0.0001 | 0.0007 | 0.0001 | 0.0014 | 0.0013 | 0.0002 |
| *Clostridium XlVb* | 0.0004 | 0.0008 | 0.0003 | 0.0005 | 0.0005 | 0.0006 | 0.0005 | 0.0003 | 0.0004 | 0.0000 | 0.0003 | 0.0008 |
| *Erysipelotrichaceae_ incertae_sedis* | 0.0004 | 0.0004 | 0.0001 | 0.0002 | 0.0002 | 0.0010 | 0.0001 | 0.0001 | 0.0001 | 0.0002 | 0.0007 | 0.0000 |
